# Risk Stratification for Early Detection of Diabetes and Hypertension in Resource-Limited Settings: Machine Learning Analysis

**DOI:** 10.2196/20123

**Published:** 2021-01-21

**Authors:** Justin J Boutilier, Timothy C Y Chan, Manish Ranjan, Sarang Deo

**Affiliations:** 1 Department of Industrial and Systems Engineering University of Wisconsin-Madison Madison, WI United States; 2 Department of Mechanical and Industrial Engineering University of Toronto Toronto, ON Canada; 3 NanoHealth NanoCare Health Services Hyderabad India; 4 Max Institute of Healthcare Management Indian School of Business Hyderabad India

**Keywords:** machine learning, diabetes, hypertension, screening, global health

## Abstract

**Background:**

The impending scale up of noncommunicable disease screening programs in low- and middle-income countries coupled with limited health resources require that such programs be as accurate as possible at identifying patients at high risk.

**Objective:**

The aim of this study was to develop machine learning–based risk stratification algorithms for diabetes and hypertension that are tailored for the at-risk population served by community-based screening programs in low-resource settings.

**Methods:**

We trained and tested our models by using data from 2278 patients collected by community health workers through door-to-door and camp-based screenings in the urban slums of Hyderabad, India between July 14, 2015 and April 21, 2018. We determined the best models for predicting short-term (2-month) risk of diabetes and hypertension (a model for diabetes and a model for hypertension) and compared these models to previously developed risk scores from the United States and the United Kingdom by using prediction accuracy as characterized by the area under the receiver operating characteristic curve (AUC) and the number of false negatives.

**Results:**

We found that models based on random forest had the highest prediction accuracy for both diseases and were able to outperform the US and UK risk scores in terms of AUC by 35.5% for diabetes (improvement of 0.239 from 0.671 to 0.910) and 13.5% for hypertension (improvement of 0.094 from 0.698 to 0.792). For a fixed screening specificity of 0.9, the random forest model was able to reduce the expected number of false negatives by 620 patients per 1000 screenings for diabetes and 220 patients per 1000 screenings for hypertension. This improvement reduces the cost of incorrect risk stratification by US $1.99 (or 35%) per screening for diabetes and US $1.60 (or 21%) per screening for hypertension.

**Conclusions:**

In the next decade, health systems in many countries are planning to spend significant resources on noncommunicable disease screening programs and our study demonstrates that machine learning models can be leveraged by these programs to effectively utilize limited resources by improving risk stratification.

## Introduction

Noncommunicable diseases, including diabetes, hypertension, and cardiovascular disease, are a global health priority [1]. Noncommunicable diseases disproportionally affect low- and middle-income countries, wherein more than 75% of all noncommunicable disease deaths (~31 million per year) occur, including over 16 million annual deaths in adults between the ages of 30 years and 69 years [1]. India faces the largest burden of noncommunicable diseases in the world with an estimated 73 million reported with diabetes and over 400 million people reported with hypertension [2,3]. Moreover, an estimated 58% of the patients with diabetes and 60%-75% of the patients with hypertension in India are undiagnosed, thereby creating a population health crisis [4]. Early detection via screening and subsequent treatment initiation can significantly reduce the burden of both diabetes and hypertension [5,6]. However, health systems in many low- and middle-income countries are already overburdened with an unfinished agenda on infectious diseases [7] and do not have enough capacity to conduct national-level noncommunicable disease screening programs [8].

Community-based screening programs can be leveraged to augment the capacity of the existing health systems by using community health workers (with limited training to conduct diabetes and hypertension screening) [9]. Owing to the large number of undiagnosed patients and the lack of awareness of noncommunicable diseases, community health workers typically conduct door-to-door and camp-based (ie, a tent staffed with community health workers) screenings to identify patients with undiagnosed diabetes and hypertension and subsequently refer them to a physician for assessment.

Community-based screening programs in low- and middle-income countries typically employ risk stratification methods that have been developed in high-income countries, leading to 3 key limitations [10,11]. First, at-risk populations in low- and middle-income countries differ significantly in social, lifestyle, and genetic aspects, thereby limiting the validity of models from high-income countries [12,13]. Second, a community-based approach severely limits the amount and complexity of data that can be collected by community health workers. Consequently, many models from high-income countries, which rely on advanced data (eg, triglyceride levels for diabetes [14]), are not applicable. Third, models from high-income countries are often calibrated to estimate the long-term risk of developing the disease [11,15] (eg, 2-10 years) rather than identifying the short-term risk of developing the disease. Owing to these limitations, application of approaches from high-income countries to community-based screening programs in low- and middle-income countries can result in poor risk stratification accuracy, reduced screening program yield, and increased cost per case identified [16].

In this study, we developed new risk stratification algorithms that are tailored for community-based screening programs in low- and middle-income countries with limited screening data. In particular, we used data collected by community health workers in Hyderabad, India and developed risk stratification models to estimate the short-term (2-month) risk for both diabetes mellitus and primary hypertension. We compared our results with several approaches from the literature, including previously developed risk scores from the United States and the United Kingdom. We also analyzed the trade-off between model accuracy and data availability by quantifying the incremental value of each data type collected during screening. Lastly, we quantified the expected reduction in the number of patients incorrectly stratified and the expected cost of incorrect risk stratification per patient.

## Methods

### Study Setting

Our study was based in Hyderabad, the capital of the state of Telangana and the fourth largest city in India with a population of 7 million [17]. Hyderabad has more than 1.7 million people living in 1400 urban slums [18]. Our catchment area included 52 urban slums and the surrounding communities. This population comprises individuals working as drivers, daily wage earners, domestic helpers, vendors, and self-employed professionals in the unorganized sector of the economy. The average family income of these residents ranges between INR 15,000 and INR 30,000 per month, which is equivalent to US $200-US $400, while the median income in Hyderabad is INR 25,000 (US $1=INR 75) per month [19].

### Data Collection

We obtained retrospective data from a social enterprise based in Hyderabad that provides screening and management of diabetes and hypertension for low-income households. These data were collected through door-to-door and camp-based screenings conducted in the urban slums of Hyderabad between July 14, 2015 and April 21, 2018 by community health workers. The door-to-door screenings were conducted in low-income areas throughout Hyderabad. In our context, “camp-based” refers to individuals that were screened at a “screening camp”—a tent staffed with community health workers and setup in a low-income area. These “screening camps” were conducted in the same locations that community health workers conducted door-to-door screenings and allowed individuals to present themselves for screening.

Each community health worker was equipped with a “Doc-in-the-Bag” kit that included a weighing scale, measuring tape, blood glucose monitor, and blood pressure/heart rate cuffs (see Figure S1 of Multimedia Appendix 1 for photographs of the kit). A mobile tablet was used to record patients’ responses to the questionnaire about family history, lifestyle, demographics, symptoms of common ailments, and to record certain anthropometric measurements and vitals (see the Data Description or Screening Questionnaire sections in Multimedia Appendix 1 for more details).

We included all individuals who visited a physician within 2 months following the screening to be assessed for a diagnosis of diabetes and hypertension. Hypertension was diagnosed based on 2 physician visits by using the JNC-VII (Seventh Report of the Joint National Committee on Prevention, Detection, Evaluation, and Treatment of High Blood Pressure) definition of hypertension [2]. Diabetes was diagnosed based on one of the following criteria: glycated hemoglobin (HbA_1c_) higher than 6.5% (48 mmol/mol) or fasting blood glucose level higher than 126 mg/dL (7 mmol/L) [10].

### Data Analysis

We developed separate models to estimate the risks for diabetes and hypertension. In each model, the outcome variable (the target) was a binary variable, indicating that a physician made a positive disease diagnosis and the predictor variables (features) were obtained from the screening data. Our primary analysis focused on determining the best models for predicting diabetes and hypertension risk and compared these models to previously developed risk scores from the United States and the United Kingdom. We compared the performance of 5 commonly used risk prediction models: decision trees, regularized logistic regression, K-nearest neighbors, random forest, and AdaBoost decision trees. We compared the performance of these models with several baseline approaches from the United States and the United Kingdom (see Table S2 of Multimedia Appendix 1). For each baseline, we calculated 3 variants (where applicable): (1) the original version based on a regression model with parameters derived from the original dataset, (2) an approximate score-based version that is intended for ease of computation by patients and providers, and (3) a version of the original regression model retrained using our Hyderabad data. In total, we considered 6 baseline models for diabetes (2 original, 2 score-based, and 2 retrained) and 2 baseline models for hypertension (1 original and 1 retrained). The hypertension model that we considered did not have an approximate score-based version (see the Baseline Approaches section in Multimedia Appendix 1 for more details).

In line with common practice, we used disjoint training (train the model), validation (tune the hyperparameters), and testing sets (test the model). We used a 10-fold cross-validation procedure to partition our data into training and testing sets. We then used 3-fold cross validation on the training set to choose our hyperparameters (see the Hyperparameter Tuning section in Multimedia Appendix 1 for more details). Once the final hyperparameters were selected, we applied the final model to the test set (that was not used as part of the model selection or fitting process in any way) to estimate generalization. We repeated the entire process 25 times to obtain more robust estimates and error bars.

For each model, we varied the discriminant threshold applied to the test set, calculated the resulting true and false positive rates, plotted them in the form of a receiver operating characteristic curve, and calculated the area under the receiver operating characteristic curve (AUC), which we used as a metric to compare different models. In total, we generated 250 test sets (25 repetitions * 10-fold) receiver operating characteristic curves for each model.

As a secondary analysis, we compared the performance of our risk stratification models, each trained with 5 different feature sets, where each set incrementally adds measurements obtained using an additional device: (1) only the questionnaire (no device measurements), (2) questionnaire and weight (weighing scale), (3) questionnaire, weight, height, and waist circumference (weighing scale and tape measure), (4) questionnaire, weight, height, waist circumference, blood pressure, and heart rate (weighing scale, tape measure, and blood pressure/heart rate cuffs), and (5) questionnaire, weight, height, waist circumference, blood pressure, heart rate, and blood glucose (weighing scale, tape measure, blood pressure/heart rate cuffs, and glucometer).

Finally, we performed a cost analysis to estimate the expected cost of incorrect risk stratification per screening for both diabetes and hypertension. We relied on previous research to estimate the cost of false positives, the cost of false negatives, and disease prevalence in India for both diabetes and hypertension. We also conducted a sensitivity analysis on each component used to estimate the expected cost of incorrect risk stratification per screening (see the Cost Analysis section of Multimedia Appendix 1 for more details).

### Statistical Analysis

For all model comparisons, we conducted a 2-sided Wilcoxon signed rank test [20] (with a significance level of .05) to check whether the medians of the AUC distributions (or cost distributions) of the 2 models were different from each other. All models and statistical tests were implemented using Python 3.5, SciPy package, and the Scikit-learn package [21]. The data and source code that support the findings of this study are available from the corresponding author.

## Results

### Data Summary

A total of 51,474 individuals were screened between July 14, 2015 and April 21, 2018. Of these individuals, 2278 (4.6%) visited a physician within 2 months following the screening (see Figure S2 of Multimedia Appendix 1). Table S1 (Multimedia Appendix 1) displays the summary statistics for individuals who did and did not visit a doctor. Table 1 displays the summary statistics of all 2278 individuals in the final data set grouped by outcome. The average age was 50.6 years and 62% (1410/2278) of the patients were female. Both random blood glucose and blood pressure were notably high with averages of 167.8 mg/dL and 145/93 mmHg across all individuals, respectively.

**Table 1:** Summary of individual screening data (N=2278).^a^

Characteristic	Diabetes diagnosis		Hypertension diagnosis		All (N=2278)
	Positive (n=833)	Negative (n=1445)	Positive (n=1676)	Negative (n=602)	
Female, n (%)	496 (59.5)	914 (63.3)	1043 (62.2)	367 (61.0)	1410 (61.9)
Age (years), mean (SD)	52.9 (11.8)	49.3 (14.0)	52.3 (13.0)	45.8 (12.9)	50.6 (13.6)
Height (m), mean (SD)	1.6 (0.1)	1.6 (0.1)	1.6 (0.1)	1.6 (0.1)	1.6 (0.1)
Weight (kg), mean (SD)	66.5 (13.1)	62.1 (12.9)	64.5 (13.2)	61.5 (12.7)	63.7 (13.1)
BMI (kg/m²), mean (SD)	27 (5.4)	25.1 (5.4)	26.2 (5.5)	24.7 (5.2)	25.8 (5.5)
Waist circumference (cm), mean (SD)	93 (11.9)	90.4 (10.3)	91.9 (11.2)	90 (10.0)	91.4 (10.9)
Heart rate (per minute), mean (SD)	88 (12.2)	84 (11.6)	86 (12.1)	86.3 (11.2)	86.1 (11.9)
Random blood sugar (mg/dL), mean (SD)	233.6 (99.2)	129.9 (44.2)	166.7 (85.3)	171 (86.6)	167.8 (85.6)
Systolic blood pressure (mmHg), mean (SD)	144.4 (22.5)	144.6 (21.0)	147.6 (21.4)	136 (19.8)	144.6 (21.6)
Diastolic blood pressure (mmHg), mean (SD)	91.1 (12.2)	93.5 (12.0)	93.6 (12.0)	89.8 (11.8)	92.6 (12.1)
Urinations per night, mean (SD)	2 (1.2)	1.5 (0.9)	1.8 (1.1)	1.5 (1.0)	1.7 (1.1)
Parental diabetes, n (%)	244 (29.3)	297 (20.6)	410 (24.5)	131 (21.8)	541 (23.7)
Parental hypertension, n (%)	210 (25.2)	319 (22.1)	408 (24.3)	121 (20.1)	529 (23.2)
Dizziness, n (%)	124 (14.9)	106 (7.3)	184 (11.0)	46 (7.6)	230 (10.1)
Numbness, n (%)	153 (18.4)	117 (8.1)	224 (13.3)	46 (7.6)	270 (11.9)
Dry tongue, n (%)	162 (19.4)	97 (6.7)	216 (12.9)	43 (7.1)	259 (11.4)
Chest pain, n (%)	40 (4.8)	15 (1.0)	47 (2.8)	8 (1.3)	55 (2.4)
Current smoker, n (%)	67 (8.0)	100 (6.9)	129 (7.7)	38 (6.3)	167 (7.3)
Medication,^b^ n (%)	578 (69.4)	413 (28.6)	863 (51.5)	128 (21.3)	991 (43.5)

^a^Diabetes and hypertension are doctor-reported diagnoses and correspond to our outcome (target) variable.

^b^Proportion of individuals currently using some type of medication, but no further details on type or reason for medication were collected.

### Model Performance

**Figure 1:**
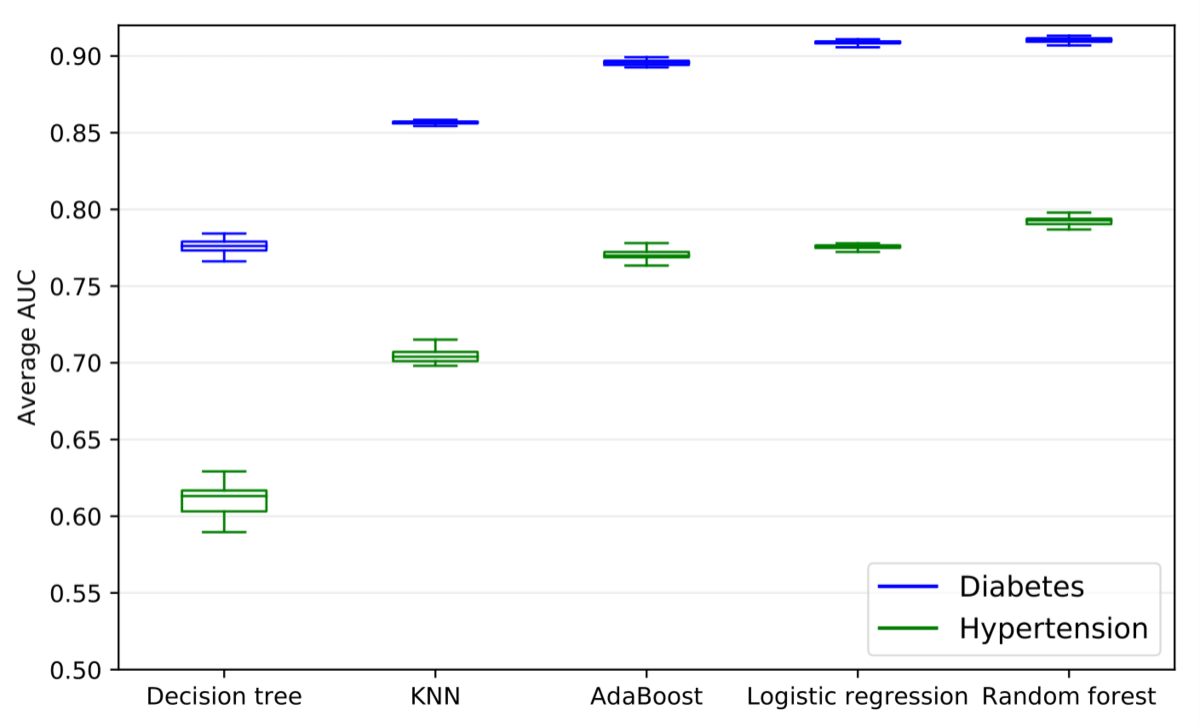
A comparison of area under the curve (AUC) distributions across 250 test sets between 5 risk stratification models for both diabetes and hypertension. The random forest model had the highest AUC for both diabetes (mean [SD], 0.910 [0.001]) and hypertension (mean [SD], 0.792 [0.002]). The upper and lower boundaries of the boxes correspond to the first and third quartiles, respectively. The line inside the box represents the median and the whiskers correspond to the minimum and the maximum of the distribution. KNN: K-nearest neighbors algorithm.

Figure 1 displays the AUC distribution across all 250 test sets of the 10 models based on 5 machine learning approaches (5 for diabetes and 5 for hypertension). For diabetes, the random forest model had the highest average AUC value (mean [SD], 0.910 [0.001]), followed by logistic regression (mean [SD], 0.909 [0.001]), AdaBoost decision trees (mean [SD], 0.896 [0.002]), K-nearest neighbors (mean [SD], 0.857 [0.001]), and decision trees (mean [SD], 0.776 [0.005]). For hypertension, random forest (mean [SD], 0.792 [0.002]) performed slightly better than logistic regression (mean [SD], 0.776 [0.001]) and AdaBoost decision trees (mean [SD], 0.770 [0.003]). K-nearest neighbors (mean [SD], 0.705 [0.004]) and decision trees (mean [SD], 0.610 [0.01]) had poorer performance. All pairwise differences were found to be statistically significant. Given these results, we focused on the random forest model when comparing with baseline approaches for both diabetes and hypertension.

**Figure 2:**
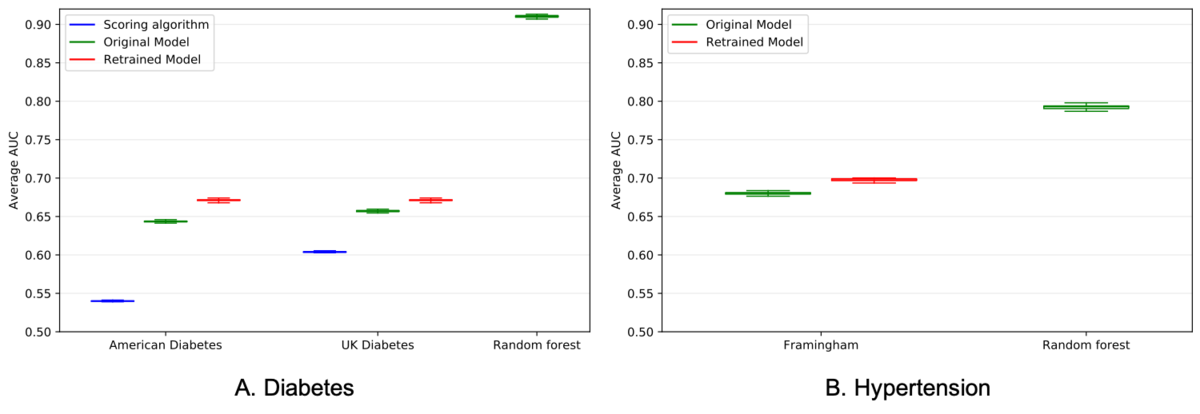
A comparison of the area under curve (AUC) distributions for the random forest model and all baseline approaches. A. For diabetes, the AUC of the random forest model improved upon the best baseline approach (UK diabetes–retrained) by 0.239 (0.910 vs 0.671, *P*<.001). B. For hypertension, the AUC of the random forest model improved upon the best baseline approach (Framingham-retrained) by 0.095 (0.792 vs 0.697, *P*<.001).

Figure 2 displays the AUC distribution for the random forest model and all baseline approaches for both diabetes and hypertension. For diabetes, the American Diabetes Association (ADA)–retrained (mean [SD], 0.671 [0.032]) and UK Diabetes–retrained (mean [SD], 0.671 [0.032]) performed best, followed by the UK–original model (mean [SD], 0.657 [0.031]) and the ADA–original model (mean [SD], 0.643 [0.033]). The ADA-scoring (mean [SD], 0.540 [0.021]) and UK-scoring (mean [SD], 0.604 [0.031]) methods performed considerably worse. For hypertension, Framingham-retrained (mean [SD], 0.698 [0.037]) performed slightly better than the Framingham–original model (mean [SD], 0.680 [0.036]). The random forest model significantly outperformed all baseline approaches. Retraining the baseline models using our data provided a statistically significant increase in their accuracy. Nevertheless, the accuracy of these retrained models was still lower than the accuracy of our random forest model.

To visualize the trade-off between false positives and false negatives, Figure S3 (Multimedia Appendix 1) displays receiver operating characteristic curves from a single randomly selected test set (out of 250) of the random forest model and baseline approaches for both diabetes and hypertension. For a fixed screening specificity of 0.9, the random forest model was able to reduce the false negative rate, on average from 0.79 to 0.17 for diabetes and from 0.72 to 0.50 for hypertension. In other words, the random forest model can reduce the number of false negatives by 620 patients per 1000 screenings for diabetes and 220 patients per 1000 screenings for hypertension.

Figure S4 (Multimedia Appendix 1) displays the normalized feature importance extracted from the random forest model. As expected, blood sugar was the most important feature for diabetes risk prediction, but several other features including many self-reported symptoms (eg, urination, dry tongue) provided predictive power. For hypertension, systolic blood pressure was the most important, followed closely by blood sugar, which is not used for hypertensive risk prediction in high-income countries, even though there is a known link between these diseases.

### Model Performance as a Function of Data Availability

**Figure 3:**
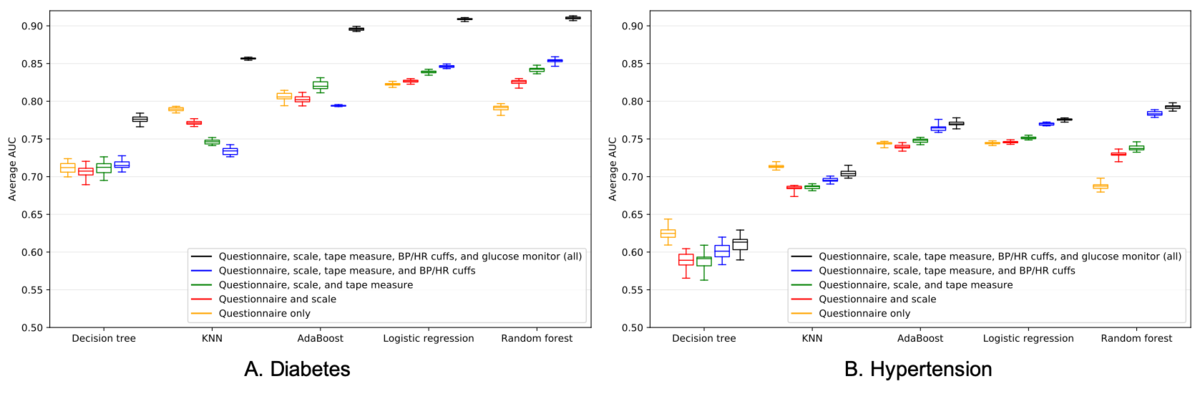
Area under the curve (AUC) distributions for 5 risk stratification models and 5 different features sets. A. Including a glucose monitor had the largest effect on diabetes risk stratification, increasing average AUC by more than 0.05 for all models. B. Including blood pressure/heart rate cuffs had the largest effect on hypertension risk stratification, increasing the average AUC by up to 0.05. BP: blood pressure; HR: heart rate; KNN: K-nearest neighbors algorithm.

Figure 3 displays the AUC distributions for the 5 risk stratification models and the 5 different features sets. For both diabetes and hypertension, a random forest model with access to only questionnaire-type features was able to capture 87% of the AUC obtained from a model with access to all features. We found that the use of a glucose monitor had the largest impact on diabetes model performance, increasing the average AUC by more than 0.05 for all models, while the use of a blood pressure/heart rate cuff had the largest impact on hypertensive risk prediction, increasing the average AUC by a mean of 0.05 across all models. See Figure S5 (Multimedia Appendix 1) for a visualization of the trade-off between false positives and false negatives.

### Cost Analysis

**Figure 4:**
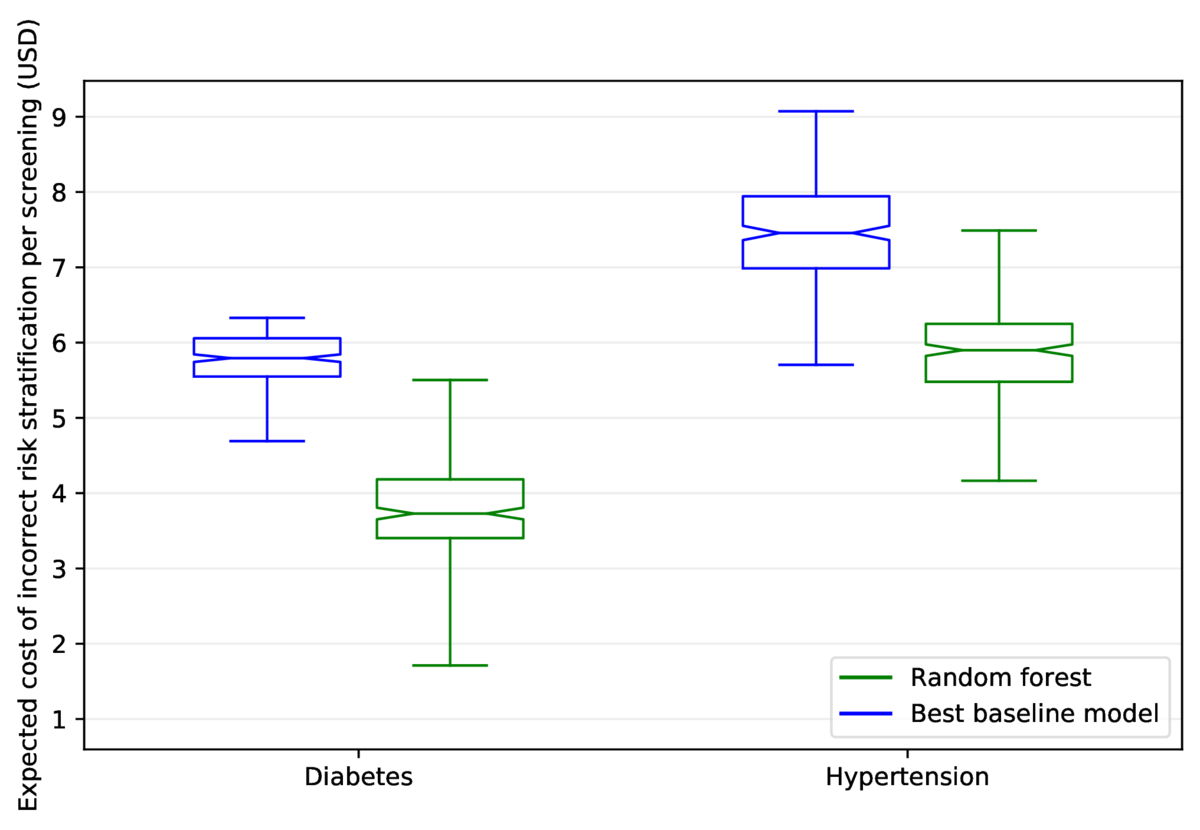
A comparison of the expected cost of incorrect risk stratification per screening across 250 test sets between the random forest and best baseline approach for both diabetes and hypertension. The random forest reduced the expected cost of incorrect risk stratification by US $1.99 per screening for diabetes (US $5.76 vs US $3.77, *P*<.001) and by US $1.60 per screening for hypertension (US $7.47 vs US $5.87, *P*<.001). The upper and lower boundaries of the boxes correspond to the first and third quartiles, respectively. The line inside the box represents the median, the whiskers correspond to the minimum and the maximum of the distribution, and the notches in the box represent the 95% confidence interval around the median.

Figure 4 displays the expected cost of incorrect risk stratification per screening for the random forest model and the best baseline approach for diabetes (UK Diabetes–retrained) and hypertension (Framingham-retrained). For the baseline models, the expected cost of incorrect risk stratification per screening was US $5.76 and US $7.47 for diabetes and hypertension, respectively. The random forest model was able to reduce the expected cost of incorrect risk stratification per screening by US $1.99 (or 35%) for diabetes and US $1.60 (or 21%) for hypertension. All cost reductions were found to be statistically significant. Figure S6 (Multimedia Appendix 1) displays the results of our sensitivity analysis.

## Discussion

This study developed risk stratification models to predict the short-term (2-month) risk in a resource-limited setting for both diabetes and hypertension. Our primary analysis demonstrated that models from high-income countries do not generalize well to the low- and middle-income countries. In particular, the random forest model had the highest prediction accuracy for both diseases and was able to outperform the best baseline approach in terms of AUC by 35.5% for diabetes and 13.5% for hypertension. Our secondary analysis found that risk stratification can be accurately performed with limited data. A random forest model with access to only questionnaire-type features was able to capture 87% of the AUC obtained from a model with access to all features, suggesting that diabetes and hypertension risk stratification can be accurately conducted in extremely resource-limited settings. Although there are circumstances where advanced measurements may be required, eliminating the need for the corresponding tools means that community health workers require less training and can travel with fewer devices.

The observed performance difference between the baseline approaches and our models can be attributed to 3 improvements. First, our models were designed for short-term risk prediction, while the baseline models were designed for long-term prediction. Even though we retrained the baseline models with our data, the features included in the models were selected for long-term prediction. For example, none of the baseline models included self-reported symptoms (eg, dry tongue, urination), which may be more suitable for short-term prediction. Second, our models include additional features not used by the baseline approaches that may provide additional insight into the social, lifestyle, and genetic differences in the population. For example, none of the risk scores from high-income countries use self-reported symptoms or random blood glucose. Although random blood glucose is not typically used in high-income settings where HbA_1c_ is preferred, it is often captured by community-based screening programs due to its operational simplicity (eg, no fasting required). For diabetes, random blood glucose was the most important feature and increased the AUC by 0.13, while for hypertension, random blood glucose was the second most important feature (see Figure S4 of Multimedia Appendix 1) and also led to an AUC increase. Third, we believe that the advanced machine learning models allowed us to extract maximum value from the small sample size and simple features available to us, whereas simple models with advanced features and large data sets may be equally effective in high-income settings.

As a by-product of our analysis, we externally validated the previously developed baseline approaches by using India-specific data. Although many of these models have been externally validated in a variety of settings, they have not been compared using India-specific data [10,11]. For example, the Framingham model for hypertensive risk has been validated in 7 countries with an average AUC of 0.80 (range 0.73-0.84) [11]. Our results show that the model is not as effective in India, where it had an average AUC of 0.70 after being retrained using local data. It is challenging to determine why the model performed poorly, but we believe that it may be due to subtle differences in the at-risk population, which manifest in the features selected by the model. Overall, our validation and comparison of baseline models highlights the importance of developing risk prediction models specifically for the low- and middle-income countries of interest.

The translation of our findings to the design and implementation of nation-wide screening programs must carefully consider costs, field accessibility, and disease management. The results of our secondary analysis indicate that the most impactful features (blood glucose, blood pressure, and heart rate) are measured using the most expensive field equipment (glucose monitor and blood pressure/heart rate cuffs). Even though these devices are more expensive, we find that including glucose monitors for diabetes screening and heart rate/blood pressure cuffs for hypertension screening can reduce the expected cost of incorrect risk stratification by US $1.35 and US $0.70, respectively (see Figure S7 of Multimedia Appendix 1). A formal cost-effectiveness analysis is needed to determine whether the gain in accuracy (and subsequent reduction is risk stratification cost) is worth the capital investment required to purchase glucose monitors and heart rate/blood pressure cuffs in low-resource settings.

There is also an important cost-tradeoff between a high false positive rate and a high false negative rate, which is determined by the discriminant threshold used to stratify patients into risk categories. Research suggests that the financial cost of a false positive is minimal (US $7 for diabetes and US $15 for hypertension) compared to that of a false negative (US $288 for diabetes and US $45 for hypertension) [22]. Our results demonstrated that the random forest model can reduce the number of false negatives by 620 patients per 1000 screenings for diabetes and 220 patients per 1000 screenings for hypertension. Extrapolating these results to a nationwide screening program in India that screens 600 million people [23] could save approximately US $1.19 billion for diabetes and US $960 million for hypertension by reducing the false negatives. In the next decade, the central government of India is planning to spend significant resources on noncommunicable disease screening programs [8] and our models can be leveraged by these screening programs to effectively utilize limited resources by improving risk stratification accuracy.

Despite the complex nature of our models, they can be easily implemented and computed into handheld tablets (or other mobile health devices) carried by community health workers without the need for a simplified, hand-computable risk score, which means we can provide the most accurate prediction without any extra effort or calculations by the community health workers. Furthermore, mobile health applications have demonstrated the ability to increase access to health care for low-income populations and to improve the capacity of the existing health systems [24]. Future research is needed to understand how to best integrate and present the risk stratification results into the community health worker workflow.

It is important to note that screening is only the first step to reducing the burden of noncommunicable diseases. Once high-risk patients are identified, they need to be linked to appropriate care and put on a disease management plan [25]. Linking patients to care and initiating disease management is a nontrivial process and governments need to carefully design nationwide disease management plans because otherwise, screening programs are unlikely to have the desired impact. Therefore, an important direction for future research includes studying the effect of screening programs on population health outcomes in the presence of current and enhanced linkages to care and disease management plans.

Our work has several limitations. First, we did not have access to an external validation set from a different study population (eg, rural slums, different state or country) to test our models. Second, our data displays a clear selection bias toward sicker patients visiting a physician within 2 months (see Table S1 of Multimedia Appendix 1). From a risk stratification perspective, the selection bias toward sicker individuals makes the problem more difficult because the model must discriminate between similar individuals. In other words, we need to identify those who actually have diabetes or hypertension from a pool of individuals who all appear to be at high risk. Finally, the differences in disease prevalence and overall health between our sample and the National Family Health Survey, Hyderabad suggest that, if applied broadly, our model may experience data shifting, which occurs when the training data differs from the application data [26]. See Table S3 of Multimedia Appendix 1 for a comparison of our data sample with the urban sample from India’s National Family Health Survey. Data shifting can negatively impact accuracy (similar to how the models from the United States and the United Kingdom performed poorly in India) and future research is needed to test our models in other settings.

In conclusion, this study found that a machine learning–based risk stratification model tailored to data collected by community-based screening programs can significantly improve risk stratification accuracy for both diabetes and hypertension in low-resource settings. Researchers and international organizations have proposed machine learning as a game changer in global health, [27-29] but there is limited documented evidence that machine learning can be effectively utilized in the resource-limited settings indicative of global health projects [30]. This study adds evidence to support machine learning in global health by quantitatively demonstrating the benefit of using these models in a novel resource-limited context.

## Appendix

Multimedia Appendix 1 Supplementary data.

## Abbreviations

ADA: American Diabetes Association
AUC: area under the receiver operating characteristic curve
HbA_1c_: glycated hemoglobin
