# Supplementary material for: Risk Stratification for Early Detection of Diabetes and Hypertension in Resource-Limited Settings: Machine Learning Analysis
Source: J Med Internet Res. 2021 Jan 21;23(1):e20123. doi: 10.2196/20123 (PMC7862003; doi:10.2196/20123)
Supplement: Multimedia Appendix 1 [file jmir_v23i1e20123_app1.pdf]

Multimedia Appendix 1

Materials and Methods

Data description

Age and gender were determined from an official government identification. Body mass index (BMI) was automatically calculated as body weight (in kilograms) divided by the square of height (in meters). Waist circumference was measured in centimeters at the umbilicus with the subject standing. Blood pressure and pulse-rate were measured on the left arm of seated participants using standard protocol [1] and a combined blood pressure / pulse-rate cuff. Random blood glucose was measured using a blood glucose monitor. Patients self-reported parental hypertension or diabetes. Patients also reported if they are currently on any medication (as a yes/no, with no further details), their current smoking status, and symptoms such as dizziness, dry tongue, foot numbness, heart-aches, and frequent nocturnal urinations. See Table S3 for a comparison of our data with India's National Family Health Survey – Hyderabad [2]. See the Screening Questionnaire section for a complete list of all screening questions.

Baseline approaches – diabetes

We considered two previously developed baseline approaches. The American Diabetes Association risk score is based on a logistic regression model with six features (age, sex, diabetic family history, hypertension, BMI, physical activity) [3] Our screening questionnaire did not include information regarding physical activity, but, because more than 75% of the Indian population is physically inactive [4], we manually assigned each patient a value of 1 for physically activity (i.e., little to no activity). We acknowledge that this is a limitation of our data. The Diabetes UK risk score is based on a logistic regression model with seven features [5], all of which were included in our screening questionnaire (age, sex, BMI, South Asian ethnicity, hypertension, diabetic family history). Furthermore, the Diabetes UK model is the only method we considered that includes a feature specifically for South Asian ethnicity.

Baseline approaches – hypertension

We considered one previously developed baseline approach. The Framingham model for hypertensive risk is based on a Weibull regression model with seven features [6], all of which were included in our screening questionnaire (age, sex, BMI, smoker, hypertensive family history, blood pressure). We used logistic regression instead of Weibull because we have only one follow-up at two months.

Hyper-parameter tuning

Initially, as part of 10-fold cross validation, we partitioned the data into two sets: set1 and the test set. Set1 comprised 90% of the data, while the test set comprised 10%. We then split set1 into two sets: a training set and a validation set. Each instance (i.e., training set) that a machine learning model is trained, we employ a 3-fold cross validation subroutine to determine the best hyper-parameters. We use average AUC across the 3 folds as our accuracy measure. We consider the following hyper-parameters via grid search. For decision trees, we optimize over the type of split (random or best) and the node splitting criteria (entropy or Gini). For regularized logistic regression, we optimize over the type of regularization (L1 or L2), the class weight (equal or balance), and the regularization parameter (0.1, 0.5, 1, 2, 5). For KNN, we optimize over the number of neighbours (10, 25, 50, 75, 100), the Minkowski distance parameter (1 or 2), and the neighbour weight (equal or distance-based). For random forest, we optimize over the number of trees (100, 250, 500, 750, 1000) and the node splitting criteria (entropy or Gini). For AdaBoost trees, we optimize over the number of trees (100, 250, 500, 750, 1000) and the learning rate (0.001, 0.01, 0.1). All other model parameters are set to default values (see the Scikit-learn package documentation for details). Our training and testing scheme allowed each training set to yield a different set of optimal hyper-parameters due to the 3-fold cross validation sub-routine. As a result, we do not report the final hyper-parameter values for each model. This approach was used to mimic application, where we would train our model / determine our hyper-parameters using a training set, and apply the final model in practice.

Cost analysis

We rely on previous research to estimate the cost of false positives, the cost of false negatives, and disease prevalence in India for both diabetes and hypertension. The cost of a false positive for screening programs is typically assumed to be equal to the cost of a follow-up clinical diagnosis, while the cost of a false negative has been estimated to be equal to 10% of the 3-year direct and indirect costs associated with the disease [7-8]. For each point on the ROC curve (i.e., discriminant threshold), we compute the *expected cost of incorrect risk stratification* as follows:

$$\text{Expected cost of incorrect risk stratification} = C\_FP * FPR * (1-P) + C\_FN * FNR * P,$$

where *C_FP* is the cost of a false positive, *C_FN* is the cost of a false negative, *FPR* is the false positive rate, *FNR* is the false negative rate, and *P* is the disease prevalence in the population. FNR and FPR are determined by the discriminant threshold, and *C_FP, C_FN,* and *P* are estimated below. Each point on the ROC curve is associated with an expected cost of incorrect risk stratification; we chose the threshold associated with the minimal cost to represent that ROC curve. We then compared the distribution of all 250 minimal cost values (one for each of the 250 test sets) between the best machine learning model and the best baseline approach.

We estimate the costs for diabetes and hypertension as follows. Note that previous research has estimated that the false negative cost is 10% of the 3-year cost of disease [7-8]. For diabetes, research has estimated that the total direct and indirect costs in India were $314 USD for a 6-month period in 2012 [9-10]. Accounting for inflation, we estimate the 2018 3-year cost of diabetes to be $2,880 USD and the cost of a false negative to be $288 USD. The cost of a false positive for diabetes is assumed to be equal to the cost of an Hb1Ac test, which is roughly $7 USD in India. The prevalence of diabetes in India is estimated to be 9% [11]. For hypertension, research has estimated that the total direct and indirect costs in India were $97.68 per year in 2012 [12]. Accounting for inflation, we estimate the 2018 3-year cost of hypertension to be $446.70 USD and the cost of a false negative to be $44.67 USD. The cost of a false positive for hypertension is assumed to be equal to the cost of two follow-up visits to measure blood pressure, which is roughly $15 USD in India. The prevalence of hypertension in India is

estimated to be 25% [13]. We conducted a sensitivity analysis on *C_FP, C_FN* and *P* for both diabetes and hypertension where we varied these values by +/- 25% and +/- 50%.

Tables and Figures

Table S1. Summary of screening data for individuals who did and did not visit a doctor.

Characteristic	Visited a physician	Did not visit a physician
Number of patients	2278	49196
Female (%)	61.9	65.4
Age, yr. (mean ± stdev)	50.6 ± 13.6	43.4 ± 13.7
Height, m. (mean ± stdev)	1.6 ± 0.1	1.6 ± 0.1
Weight, kg. (mean ± stdev)	63.7 ± 13.1	60.4 ± 13.1
BMI, kg/m² (mean ± stdev)	25.8 ± 5.5	24.7 ± 5.2
Waist circumference, cm. (mean ± stdev)	91.4 ± 10.9	86.7 ± 11.2
Heart rate, per min. (mean ± stdev)	86.1 ± 11.9	84.6 ± 12.4
Random blood sugar, mg/dL (mean ± stdev)	167.8 ± 85.6	133.3 ± 61.6
Systolic BP, mmHg (mean ± stdev)	144.6 ± 21.6	126.2 ± 21.1
Diastolic BP, mmHg (mean ± stdev)	92.6 ± 12.1	83.0 ± 12.7
Urinations per night (mean ± stdev)	1.7 ± 1.1	1.7 ± 1.2
Parental diabetes (%)	23.7	18.3
Parental hypertension (%)	23.2	21.1
Dizziness (%)	10.1	15.5
Numbness (%)	11.9	15.7
Dry tongue (%)	11.4	11.7
Heart ache (%)	2.4	11.6
Current smoker (%)	7.3	7.9
Medication (%)	43.5	19.3

Table S2. Summary of baseline models.

Model	Original regression	Scoring algorithm	Retrained regression
Diabetes risk prediction			
American Diabetes Association	Logistic	6 features	Logistic
Diabetes UK	Logistic	7 features	Logistic
Hypertensive risk prediction			
Framingham	Weibull (7 features)	N/A	Logistic*

**We use logistic regression instead of Weibull because we have only one follow-up at two months.*

Table S3. Comparison of our data sample and the urban data sample from the National Family Health Survey of India – Hyderabad.

Characteristic		Our data		National Family Health Survey – Hyderabad	
		Male (%)	Female (%)	Male (%)	Female (%)
BMI	Below normal (<18.5 kg/m^2)	5.2	6.4	20.3	12.8
	Overweight (>25.0 kg/m^2)	53.3	52.2	33.1	48.7
Blood sugar	High (>140 mg/dl)	45.7	44.9	7.9	9.3
	Very high (>160 mg/dl)	36.9	36.6	7.0	5.9
Blood pressure	Above normal (SBP: 140-159 or DBP: 90-99)	10.3	11.0	15.3	9.4
	Moderately high (SBP: 160-179 or DBP: 100-109)	3.9	3.0	4.8	2.2
	Very high (SBP > 180 or DBP>110)	3.4	1.5	4.3	0.7

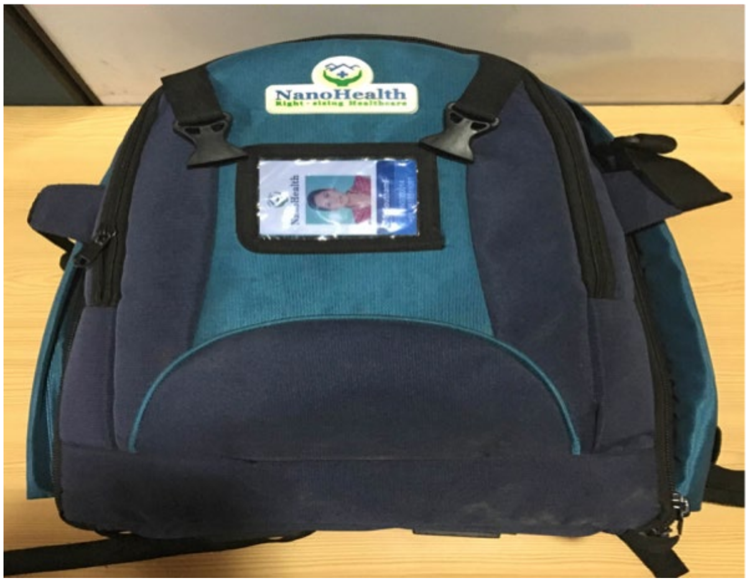

(a) outside

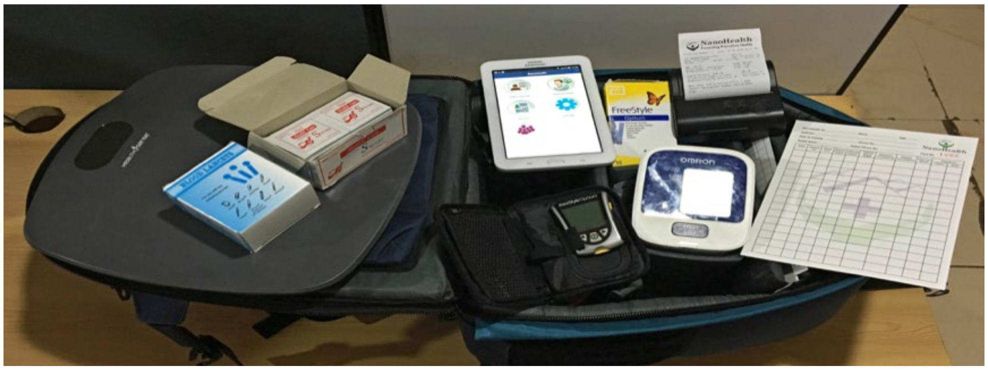

(a) contents

Figure S1. Photographs of the "Doc-in-a-Bag" kit used by NanoHealth's CHWs. (a) The outside of the backpack includes the CHW identification card. (b) The contents of the kit include a mobile tablet, weighing scale, measuring tape, blood glucose monitor, and blood pressure / heart rate cuffs.

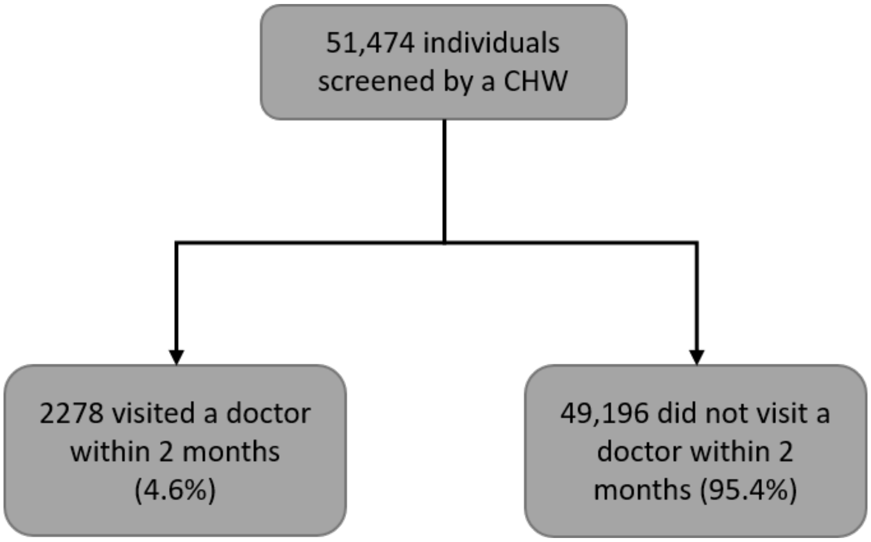

Figure S2. Study population and exclusion criteria flowchart.

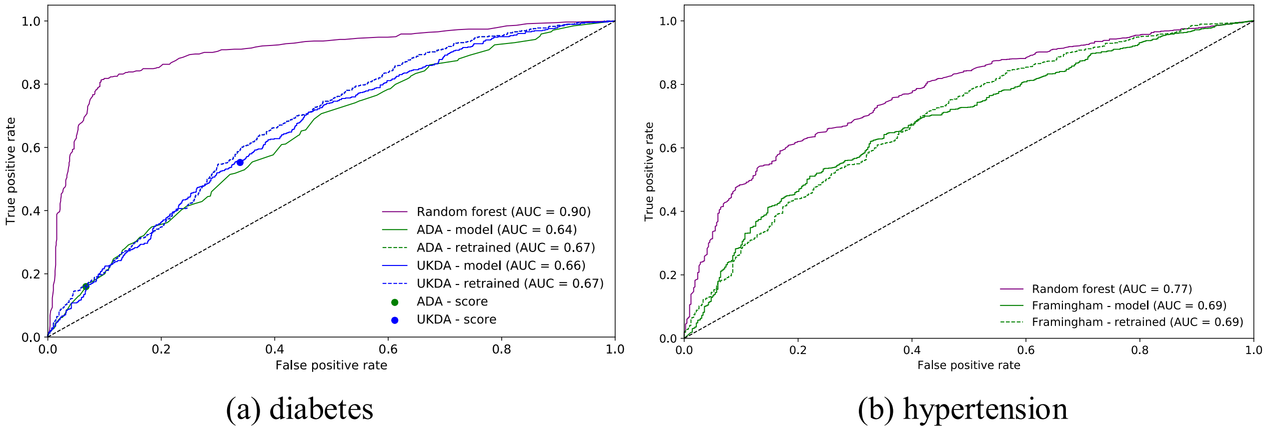

Figure S3. A comparison of ROC curves from a randomly selected test set (out of 250) between the random forest model and baseline approaches. Solid lines denote original models, dashed lines denote retrained models, and dots represent scoring algorithms. (a) The random forest model was able to produce between 7 and 61 fewer false negatives (depending on the false positive rate) as compared to UKDA – retrained. (b) The random forest model was able to produce between 0 and 19 fewer false negatives as compared to Framingham – retrained.

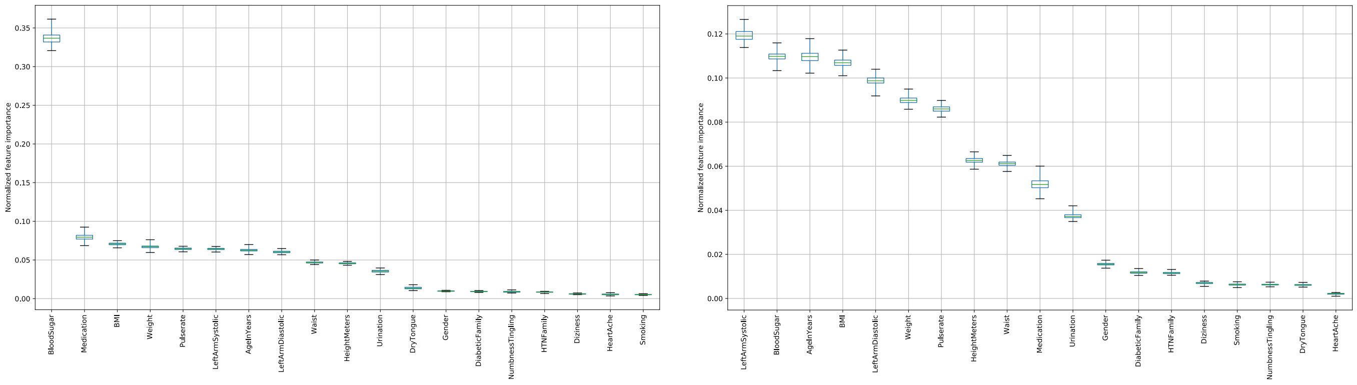

(a) diabetes (b) hypertension

Figure S4. Normalized random forest feature importance for (a) diabetes and (b) hypertension. The importance of a feature is computed as the (normalized) total reduction of the Gini criterion brought by that feature. The boxplots are used to display the distribution of feature importances across the 250 different models used to make test set predictions.

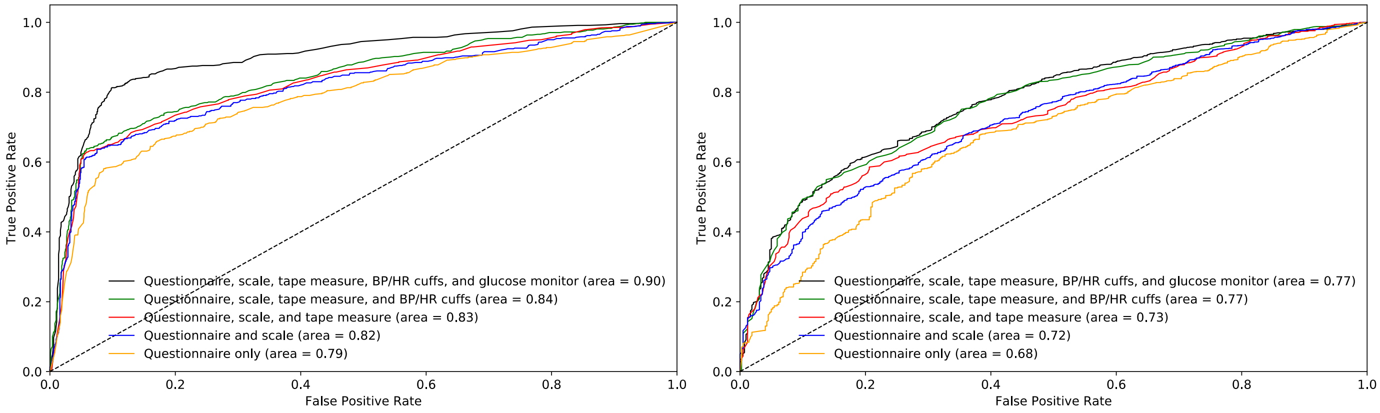

(a) diabetes (b) hypertension

Figure S5. A comparison of random forest ROC curves from a randomly selected test set (out of 250) for the five different features sets. (a) Including a glucose monitor increased the AUC for diabetes by 0.056 (0.910 vs. 0.854, P<0.0001). (b) Including blood pressure / heart rate cuffs increased AUC for hypertension by 0.045 (0.783 vs. 0.738, P<0.0001).

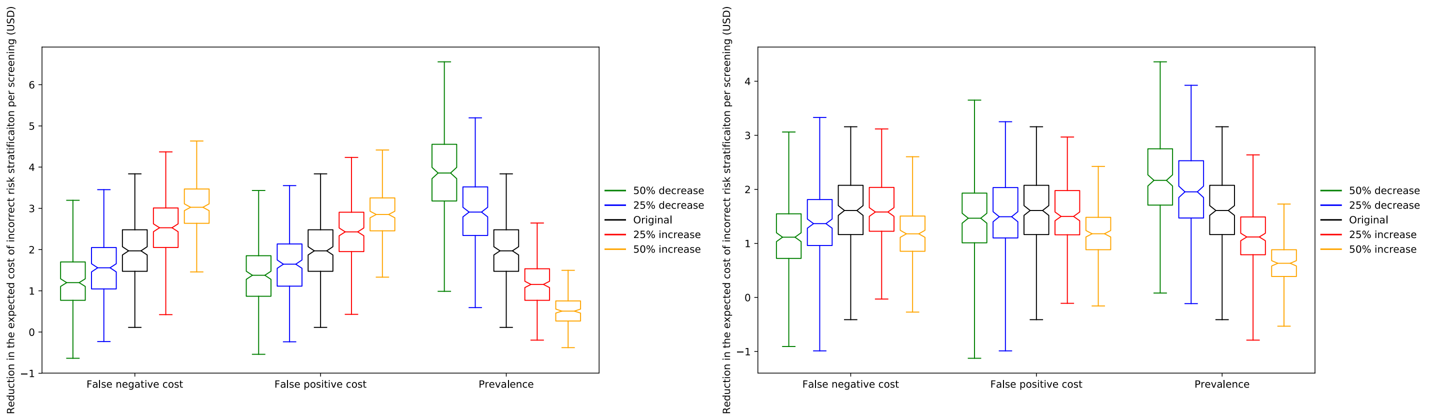

(a) diabetes

(b) hypertension

Figure S6. Sensitivity analysis for the reduction in the expected cost of incorrect risk stratification per screening. (a) Cost reductions for diabetes increased as the cost of false positives and false negatives increased and decreased as the disease became more prevalent. (b) Cost reductions for hypertension were insensitive to the cost of false positives and false negatives, and decreasing as a function of prevalence. The upper and low bounds of the boxes correspond to the first and third quartiles, respectively. The line inside the box represents the median, the whiskers correspond to the minimum and maximum of the distribution, and the notches in the box represent the 95% confidence interval around the median.

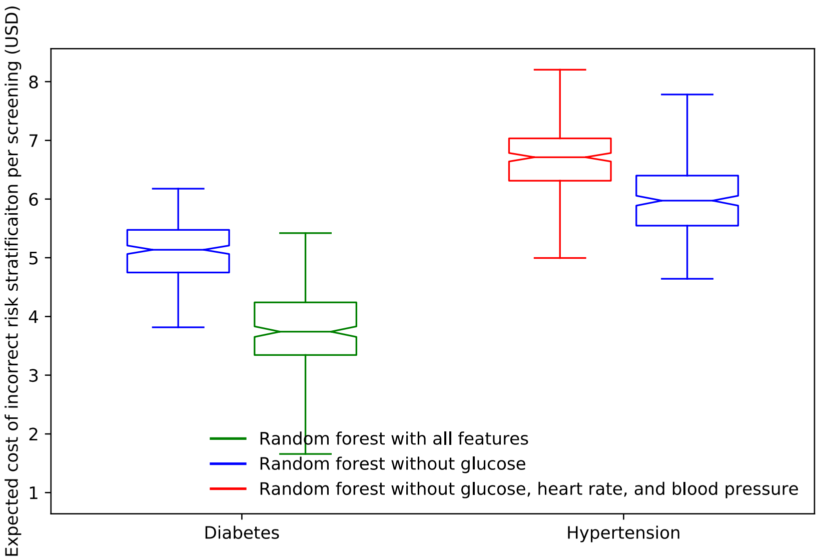

Figure S7. The expected cost of incorrect risk stratification per screening from including additional features for both diabetes and hypertension. Including random blood glucose for diabetes screening reduced costs by $1.35 per screening ($5.12 vs. $3.77, P<0.0001), while including blood pressure and heart rate for hypertension screening reduced costs by $0.70 per screening ($6.71 vs. $6.01, P<0.0001).

Screening Questionnaire

The individual questions that comprise the screening questionnaire are shown below. The questionnaire was administered in the local language and recorded by community health workers using a mobile tablet application. As a result, there is no formal paper version of the questionnaire. Although detailed demographic information was collected (including personal information), the data that we obtained was de-identified. In particular, we only obtained age (in years) and gender from the demographic data section.

Demographic data

1. ID type
2. ID Value
3. Gender
4. Date of birth (Age)
5. First name & last name
6. Mobile no
7. Email
8. Father/Spouse name
9. Address
10. What is your blood type?
11. Person Picture
12. Picture of ID Card
13. City
14. Pin Code

Personal details

1. Are you on any medication? Y/N
2. Have you recently experienced any heart aches? Y/N
3. Do you smoke tobacco? Y/N
4. Height: ______ Weight: __________
5. Waist: _______

Hypertension

1. Blood Pressure
    a. Left hand: ______
    b. Right hand: ______
2. Pulse: __________

Diabetes

1. Mother or father diagnosed with
    a. Hypertension? Y/N
    b. Diabetes? Y/N
2. Do you often feel dizzy? Y/N
3. How many times do you wake up at night for urination: _______
4. Do you often feel thirsty? Y/N
5. Do you have any numbness or tingling sensations in your hand/ feet? Y/N
6. Blood Sugar Category: Random / Fasting
    a. Blood Sugar __________

References

1. TRUE Consortium. Recommended Standards for Assessing Blood Pressure in Human Research Where Blood Pressure or Hypertension Is a Major Focus. *Kidney Int Rep*. 2017;2(4):733-738. doi:10.1016/j.ekir.2017.02.009

2. *National Family Health Survey: Fact Sheet*. Ministry of Health and Welfare, Government of India; 2016.

3. Bang H, Edwards AM, Bomback AS, et al. A patient self-assessment diabetes screening score: development, validation, and comparison to other diabetes risk assessment scores. *Ann Intern Med*. 2009;151(11):775-783.

4. Anjana RM, Pradeepa R, Das AK, et al. Physical activity and inactivity patterns in India - results from the ICMR-INDIAB study (Phase-1) [ICMR-INDIAB-5]. *Int J Behav Nutr Phys Act*. 2014;11(1):26-26. doi:10.1186/1479-5868-11-26

5. Gray LJ, Davies MJ, Hiles S, et al. Detection of impaired glucose regulation and/or type 2 diabetes mellitus, using primary care electronic data, in a multiethnic UK community setting. *Diabetologia*. 2012;55(4):959-966. doi:10.1007/s00125-011-2432-x

6. Parikh NI, Pencina MJ, Wang TJ, et al. A Risk Score for Predicting Near-Term Incidence of Hypertension: The Framingham Heart Study. *Ann Intern Med*. 2008;148(2):102. doi:10.7326/0003-4819-148-2-200801150-00005

7. Chatterjee R, Narayan KMV, Lipscomb J, et al. Screening for diabetes and prediabetes should be cost-saving in patients at high risk. *Diabetes Care*. 2013;36(7):1981-1987. doi:10.2337/dc12-1752

8. Chatterjee R, Narayan KMV, Lipscomb J, Phillips LS. Screening adults for pre-diabetes and diabetes may be cost-saving. *Diabetes Care*. 2010;33(7):1484-1490. doi:10.2337/dc10-0054

9. Akari S, Mateti UV, Kunduru BR. Health-care cost of diabetes in South India: A cost of illness study. *J Res Pharm Pract*. 2013;2(3):114-117. doi:10.4103/2279-042X.122382

10. Yesudian CAK, Grepstad M, Visintin E, Ferrario A. The economic burden of diabetes in India: a review of the literature. *Glob Health*. 2014;10:80-80. doi:10.1186/s12992-014-0080-x

11. Pradeepa R, Mohan V. Prevalence of type 2 diabetes and its complications in India and economic costs to the nation. *Eur J Clin Nutr*. 2017;71(7):816.

12. Biswas A, Singh RK, Singh SK. Medical and non-medical cost of hypertension and heart diseases in India. Halsall J, ed. *Cogent Soc Sci*. 2016;2(1):1250616. doi:10.1080/23311886.2016.1250616

13. Gupta R, Gaur K, S. Ram CV. Emerging trends in hypertension epidemiology in India. *J Hum Hypertens*. Published online September 25, 2018. doi:10.1038/s41371-018-0117-3
